# Fuzzy inference systems for mineral prospectivity modeling-optimized using Monte Carlo simulations

**DOI:** 10.1016/j.mex.2022.101629

**Published:** 2022-02-03

**Authors:** Bijal Chudasama

**Affiliations:** Information Solutions Unit – Geological Survey of Finland, Finland

**Keywords:** Fuzzy Inference Systems, Knowledge-driven modeling, Model uncertainties, Parameter optimization, Monte Carlo Simulations, Fuzzy membership functions, Confidence levels, Mineral Exploration, Target Prioritization

## Abstract

This paper uses Monte Carlo simulations to estimate the parameters of rule-based fuzzy inference systems (FISs) designed for mineral prospectivity modeling. The targeted process for the case study is gold mineralization in the Rajapalot project area in northern Finland. Mamdani-type FISs are developed and implemented for the predictive modeling of favorable structural settings and favorable chemical traps causing gold enrichment in host rocks from ore-bearing hydrothermal fluids. The parameters of the fuzzification functions control the output fuzzy membership values. Traditionally these parameters are chosen subjectively based on the expert's domain knowledge. This study uses drill core data statistics to define the distribution of the parameters. Subsequently, Monte Carlo simulations are used to simulate the corresponding fuzzy membership values and optimize the FISs.

• Capturing the complexities of the multi-processes geodynamic systems and the possible interplay mineralization-related geological aspects using ‘If-Then’ rule-based fuzzy inference systems.
• Implementation of Monte Carlo simulations for quantification of uncertainties related to a Mamdani-type FIS-based prospectivity modeling.
• Reporting prospectivity modeling results at different confidence levels for informed decision making on selection of exploration targets.

## Highlights

• Mineral prospectivity modeling at the target scale for identification of drilling targets.
• Monte Carlo simulations for optimization of fuzzy inference systems.
• Quantification of uncertainties related to the fuzzy inference systems-based mineral prospectivity modeling.
• Identification of exploration targets at varying confidence levels for informed decision making.

## Graphical abstract

**Figure:**
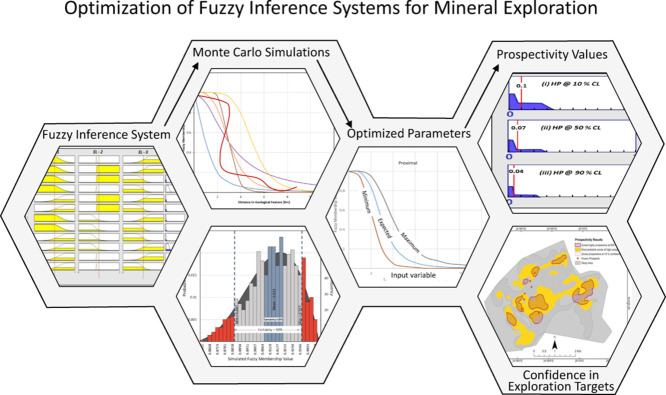

Specifications table

**Table:** 

Subject area:	Earth and Planetary Sciences
More specific subject area:	Mineral Prospectivity Modeling
Method name:	Optimization of a Mamdani-type fuzzy inference systems using Monte Carlo simulations.
Name and reference of original method:	Mamdani-type fuzzy inference systems - Mamdani and Assilian (1975). An experiment in linguistic synthesis with a fuzzy logic controller: Int. J. Man-Machine Stud., v. 7, no. 1, p. 1–13. Fuzzy inference systems applied to Mineral Prospectivity Modeling - Porwal et al. (2015). Fuzzy inference systems for prospectivity modeling of mineral systems and a case-study for prospectivity mapping of surficial Uranium in Yeelirrie Area, Western Australia. Ore Geology Reviews, 71, 839–852. Monte Carlo simulations for optimizing Fuzzy logic overlay - Lisitsin, V. A., Porwal, A., and McCuaig, T. C., 2014. “Probabilistic fuzzy logic: quantifying uncertainty of mineral prospectivity models using Monte Carlo simulations,” Mathematical Geosciences, 46(6), 747–769.
Resource availability:	Data – Proprietary Exploration Datasets from Mawson Gold Ltd. Data is confidential and cannot be released publicly. Software – This method can be implemented in any statistical data analysis software (example – Matlab, Octave, MS-Excel, XLSTAT, Oracle Crystal Ball), or using open-source scientific programming languages such as python, R. Hardware – Processor Intel(R) Core(TM) i7–8750H CPU @ 2.20 GHz, 2208 Mhz, 6 Core(s), 12 Logical Processor(s).

## Introduction

### Prospectivity modeling using a fuzzy inference system (FIS)

A fuzzy inference system (FIS) is a knowledge-driven expert system based on the concept of fuzzy sets and fuzzy logic [9], [15]. The main components of a FIS are the fuzzy membership functions and the fuzzy-*’If-Then’* rules. The output of a FIS depends primarily on the fuzzy membership values generated from the fuzzy membership functions. Traditionally, the parameters of the membership functions are identified based on domain knowledge of the geoscientist. Hence model-based uncertainties affect FIS-based prospectivity models most significantly [7,11,13,1,8]. The model uncertainty stems from the uncertainty in assigning the parameters and the type of membership functions that are used for deriving fuzzy membership values of the input evidence layers. Hence to optimize a FIS this study first utilizes the descriptive statistics of the values attained by mineralized and non-mineralized drill core locations in the evidence layers for defining the parameters of the membership functions. This was followed by Monte Carlo simulations (MCS) of the parameters and the fuzzy membership values from the probability distributions defined from data statistics.

### Proposed method - FIS optimization using Monte Carlo simulations (MCS)

The implementation of a Mamdani-type FIS-based mineral prospectivity modeling involves the following steps [12]: (1) fuzzification of the input numeric values to the degree of membership to the linguistic fuzzy sets using mathematical functions; (2) combining the linguistic values of the input variables using fuzzy operators by constructing fuzzy *‘‘If-Then’’* rules; (3) aggregation of outputs across all the *‘If-Then’* rules; and (4) defuzzification of the output aggregate to calculate the output prospectivity values and generate the prospectivity map. In the method proposed in this paper, we modify the fuzzification step at (1) by implementing the Monte Carlo simulations (MCS) technique (Heuvelink 1998, 1999; [8]). The MCS method is used to assess and quantify uncertainty in the fuzzy membership values [7,1,8]. This method was first applied to fuzzy logic overlay by Lisitsin et al. [8]. Here we extend it to the rule-based FISs where the proposed method simulates the fuzzy membership values based on the simulations of the parameters defining the membership functions. The optimized parameters are then used to design the FISs mapping the potential of the targeted mineral systems.

#### Detailed steps

**Fig. 1:**
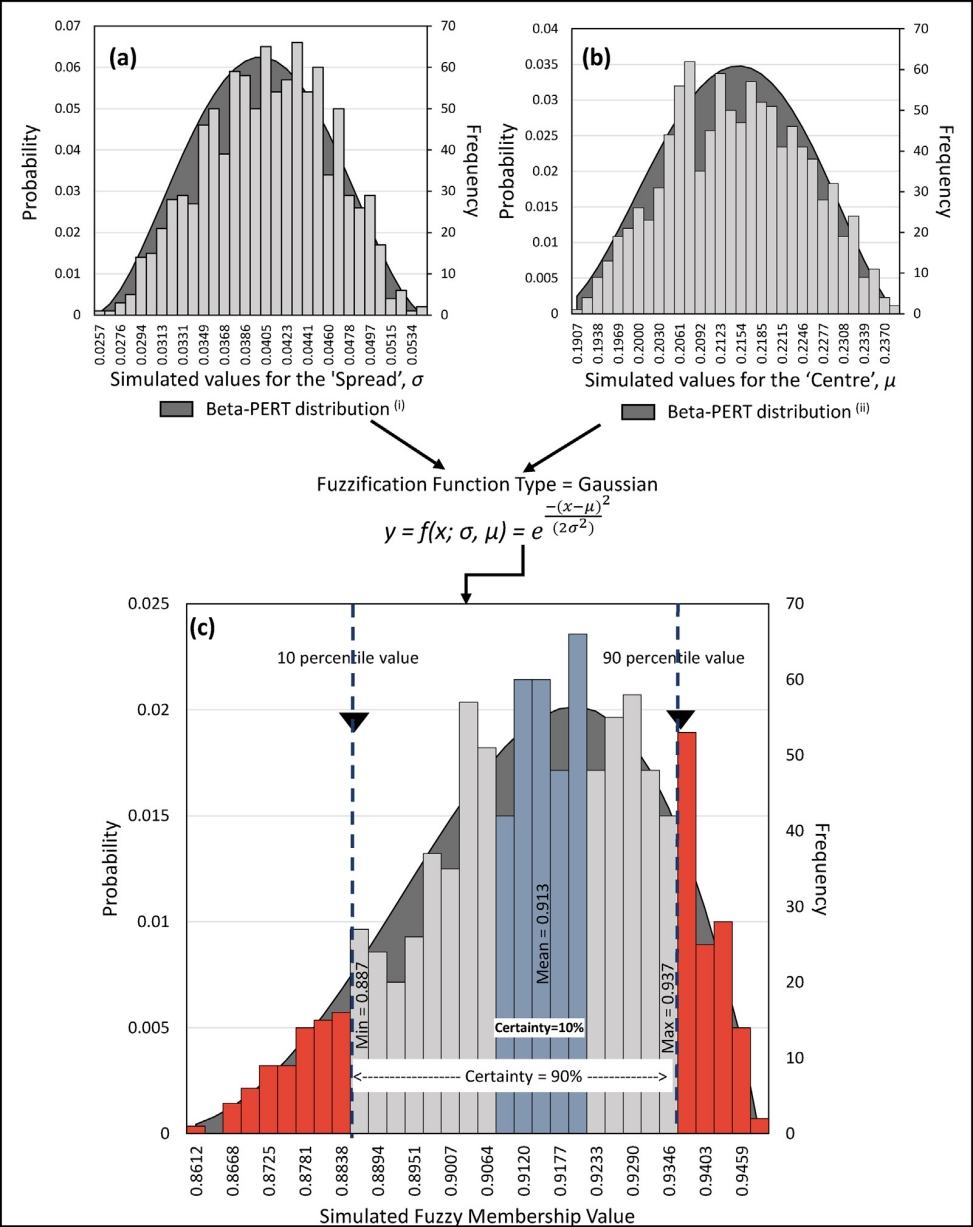
Monte Carlo simulations-based membership function parameter estimation for fuzzification of fuzzy set ‘mineralized anomalies’ in the input evidence layer representing NE-SW trending magnetic response. (a) Simulations of the spread, *σ,* parameter of the membership function from a theoretical Beta-Pert distribution. (b) Simulations of the center, *µ,* parameter of the membership function from a theoretical Beta-Pert distribution. (c) Simulation of the output fuzzy membership values from the simulated parameters.

First, the initial model parameters were assigned to conform the shape of the membership functions to the training data statistics (Table 1). These values were considered as the ‘expected’ values for the membership function parameters. Next assuming, that the parameters of the fuzzy membership functions have a beta-PERT distribution [7,8], ‘minimum’ and ‘maximum’ values were assigned to the parameters, defining the corresponding distribution curve for each of the parameters of the functions (Fig. 1a and b). The beta-PERT is a type of beta statistical distribution bounded by the minimum, maximum and the mode values [4,6,10]. With these parameters, a continuous distribution of a wide variety of shapes such as L, J, U, or a nearly-normally distributed bell-shaped curve can be defined. Beta distributions are often used when there is no training data, and the only information available is the expert knowledge about the optimistic, most likely, and pessimistic values. However, because of its flexibility, it can also be defined to model the random variables such that the distribution can approximate the variations identified in the data [10]. Hence it is applicable to modeling procedures in various fields for simulations and uncertainty assessments. Some such studies are Johnson et al. [5] for assessing potential of natural gas in the Horn River Basin in Canada; Damgaard and Irvine, [3] for exploring spatial and temporal patterns and modeling plant abundance from plant cover data; Xu and Chowdhury [14], for probabilistic analysis of structured rock and soil slopes.

**Table 1:** Statistical description of the values attained by mineralized and non-mineralized drill core sections in the evidence layers selected as inputs to prospectivity modeling.

No.	Evidence Layers:	Drill core sections	Minimum Value	Maximum Value	1st Quartile	Median	3rd Quartile	Mean	Standard deviation (n-1)	Standard error of the mean	Lower bound on mean (95%)	Upper bound on mean (95%)	Standard error (Kurtosis - Fisher)	Mean absolute deviation
1	Density of magnetic TD's 0 and +/- ∏/4 contours	Non-mineralized	0.220	0.922	0.521	0.602	0.702	0.609	0.136	0.014	0.581	0.637	0.488	0.104
		Mineralized	0.352	0.838	0.554	0.686	0.745	0.654	0.123	0.010	0.634	0.674	0.396	0.105
2	Ratio of In-phase to Quadrature components of AEM data	Non-mineralized	0.380	0.405	0.391	0.392	0.395	0.393	0.005	0.000	0.392	0.394	0.488	0.003
		Mineralized	0.366	0.395	0.390	0.392	0.394	0.391	0.004	0.000	0.391	0.392	0.396	0.003
3	NW-SE trending anomalies	Non-mineralized	0.236	0.527	0.391	0.410	0.437	0.411	0.045	0.005	0.401	0.420	0.488	0.032
		Mineralized	0.273	0.535	0.360	0.385	0.411	0.386	0.042	0.003	0.380	0.393	0.396	0.032
4	NE-SW trending anomalies	Non-mineralized	0.083	0.514	0.136	0.167	0.225	0.191	0.078	0.008	0.175	0.206	0.488	0.060
		Mineralized	0.089	0.421	0.186	0.211	0.240	0.217	0.049	0.004	0.209	0.225	0.396	0.036
5	Density of gravity worms	Non-mineralized	0.051	0.835	0.250	0.444	0.606	0.441	0.220	0.022	0.396	0.485	0.488	0.191
		Mineralized	0.036	0.838	0.586	0.700	0.811	0.682	0.136	0.011	0.660	0.704	0.396	0.112
6	Density of interpreted lithological contacts weighted by their competence- or reactivity- contrasts	Non-mineralized	0.000	0.887	0.472	0.535	0.608	0.524	0.168	0.017	0.490	0.558	0.488	0.108
		Mineralized	0.234	0.942	0.545	0.610	0.720	0.622	0.137	0.011	0.599	0.644	0.396	0.109
7	Densities interpreted structures weighted by their sinuosity OR density of structural intersection zones	Non-mineralized	0.000	0.696	0.212	0.287	0.418	0.313	0.152	0.015	0.282	0.343	0.488	0.123
		Mineralized	0.063	0.891	0.281	0.375	0.482	0.410	0.180	0.015	0.380	0.439	0.396	0.141
8	Distance to interpreted Late faults	Non-mineralized	0.000	0.783	0.068	0.108	0.151	0.138	0.146	0.015	0.108	0.167	0.488	0.088
		Mineralized	0.000	0.293	0.019	0.047	0.080	0.059	0.048	0.004	0.051	0.067	0.396	0.036

Number of observations: Mineralized drill core points: 148; Non mineralized drill core points: 96

All input data values were rescaled from 0 to 1. Abbreviations: TD - Tilt Derivative, AEM - Airborne Electromagnetics

For the current study, the bounding parameters of the beta-PERT distribution, i.e., ‘minimum’ and ‘maximum’ values were derived from the descriptive statistics of different data groups in the drill core data, for instance using the quartile 1 and quartile 3 values, respectively (Table 1). The values for each parameter were then randomly extracted 1000 times from the beta-PERT distribution defined by the ‘minimum’, ‘expected’ and ‘maximum’ values of the membership function parameters, and the corresponding output fuzzy membership values were estimated. This created a probability distribution of the output fuzzy membership values (Fig. 1c). Fig. 1a and b present the distribution of parameters and the values simulated from the distribution curve using MCS. Fig. 1c presents the output membership function values derived from the simulated parameters.

From the distribution of output membership values in Fig. 1c, we can extract the range of ‘probable’ values at varying certainty levels. For instance, the 10% certainty level gives a narrow range of the output fuzzy membership values but the range at 90% certainty is wide (Fig. 1c). Hence, at 90% certainty we can provide the least, the most probable and the highest membership values. Consequently, we use the 10-, 50- and 90-percentile membership values from the 90% certainty envelope to provide the least, the most probable and the highest membership values (Fig. 1c). The 10-percentile value is among the lowest or the most conservative estimate of the output, and hence a 90% confidence level is assigned to it. The 90-percentile value gives an estimate on the higher side of the density distribution. It is therefore the most optimistic estimate of the fuzzy membership value and hence 10% confidence level is assigned to the 90 percentile values. The 50-percentile value is the most probable estimate and hence is the ‘expected’ prediction of prospectivity values, supplemented by the10 and 90% confidence level values.

**Fig. 2:**
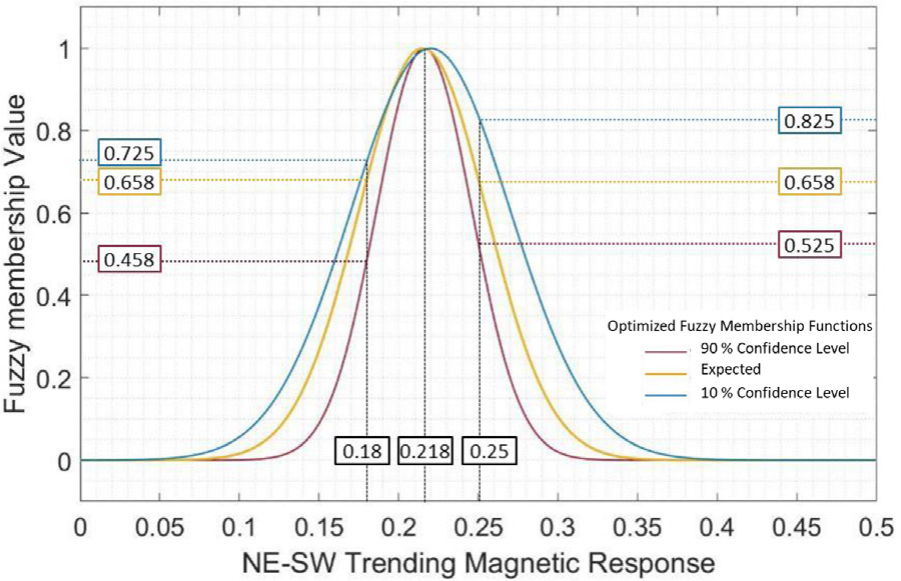
Example of optimized membership functions for the fuzzy set ‘mineralized anomalies’ in the evidence layer representing NE-SW magnetic anomalies. (Optimization process is presented in Fig. 1).

**Fig. 3:**
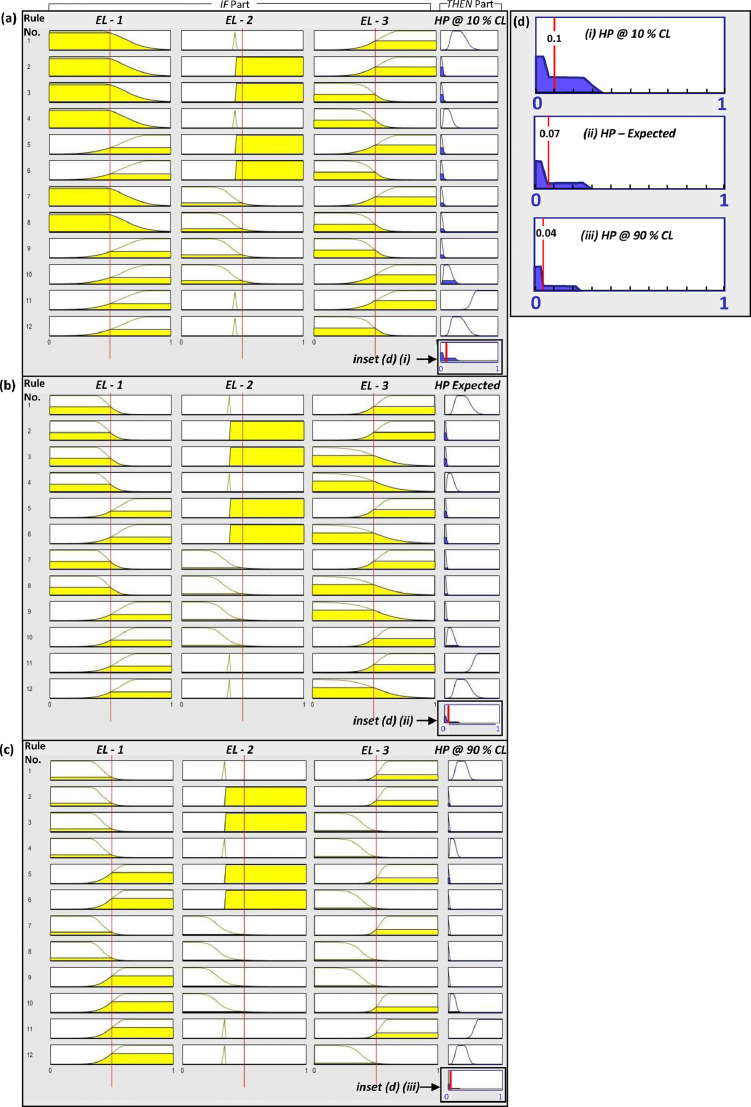
’If-Then’ rule-based structure of the FIS mapping the potential of presence of host rocks and chemical traps at (a) 10% confidence level, (b) the expected (i.e., most probable) FIS and (c) at 90% confidence level. An example for a pixel with values [0.5] in all the evidence layers EL – 1 to EL – 3 and its mapped output potential for host rock is presented at 10% -, expected and 90% - confidence levels in (a), (b) and (c), respectively. The input feature vector of the example pixel is plotted on all the 12 rules in (a) to (c). (d) Aggregate of the output fuzzy functions over all the 12 ‘If-Then’ rules for each FIS in (a), (b) and (c). For the input pixel value of [0.5] in all evidence layers EL – 1, EL – 2 and EL – 3, the output host potential reduces from 0.1 to 0.07 to 0.04, as the confidence level increases from 10% to 90% in (a) to (c), respectively. The area of the polygon diminishes and the defuzzified centroid value shifts to the left. In the ’If’-part the input values are marked by the red lines. The yellow color indicates the output fuzzy membership values for each function (the fuzzy functions are blank if the output fuzzy membership value for a given input value is 0). In the ‘Then’-part, (i.e. the output consequent membership function), the blue portion indicates the minimum implication, that is, the area of an output consequent function after it has been truncated by the firing strength (= minimum of the all fuzzy membership values in the “IF” part) of the rule. The last function in the output prospectivity column-HP, shows the aggregate of all output fuzzy functions. The red line on this function indicates the defuzzified crisp value. The insets are presented in frame (d). The abbreviations used in the Figure are: EL – 1 = Evidence Layer representing the density of magnetic TD's ‘0′ and ‘+/- ∏/4′ contours; EL – 2 = Evidence Layer representing the ratio of the in-phase to quadrature components of the airborne electromagnetic data; EL – 3 = Evidence Layer representing the density of lithological contacts weighted by either competence- OR reactivity-contrast values across the contacts; HP = Host Potential; CL = Confidence Level.

**Fig. 4:**
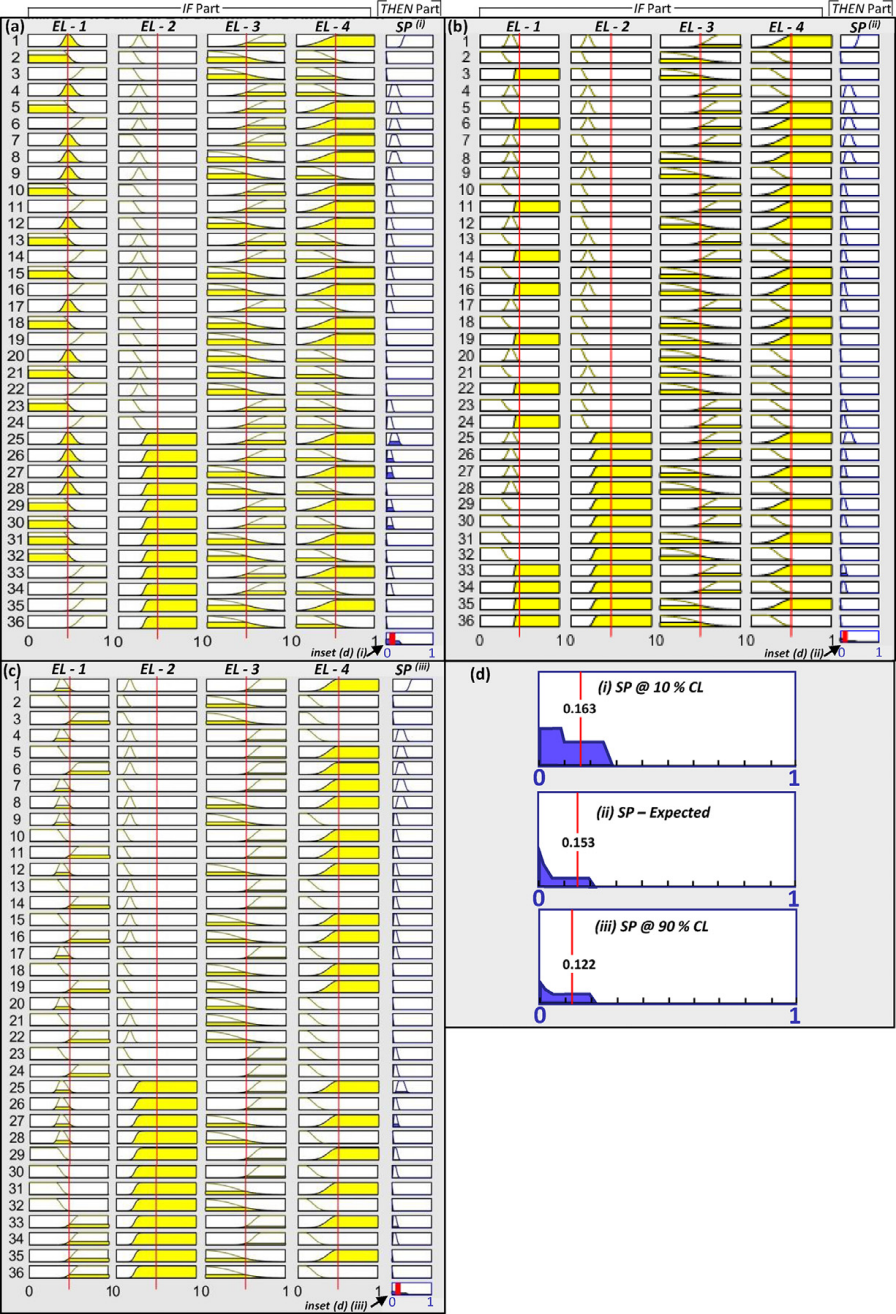
’If-Then’ rule-based structure of the FIS mapping the potential of favorable structural settings at (a) 10% confidence level, (b) the expected (i.e. most probable) FIS and (c) 90% confidence level. An example for a pixel with values [0.5] in the all the evidence layers EL – 1 to EL – 4 and it's mapped output potential for favorable structural settings is presented at 10% -, expected and 90% - confidence levels in (a), (b) and (c), respectively. The input feature vector of the example pixel is plotted on all the 36 rules. The last function in the output prospectivity column-HP, shows the aggregate of all output fuzzy functions. The red line on this function indicates the defuzzified crisp value. The insets are presented in frame (d). (d) Aggregate of the output fuzzy functions over all the 36 ‘If-Then’ rules for each FIS in (a), (b) and (c). The output potential reduces from 0.163 to 0.153 to 0.122 as the confidence level increases from 10% to 90% in (a) to (c), respectively. The area of the polygon diminishes and the defuzzified centroid value shifts to the left as seen in (d). (For the explanation of colors refer to the caption of Fig. 3). The abbreviations used in the Figure are: EL – 1 = Evidence Layer representing NW-SE trending magnetic response; EL – 2 = Evidence Layer representing NE-SW trending magnetic response; EL – 3 = Evidence Layer representing density of gravity worms; EL – 4 = Evidence Layer representing density of interpreted structures weighted by the sinuosity of the structure OR density of structural intersection zones; SP = Favorable Structural Settings Potential; CL = Confidence Level.

**Fig. 5:**
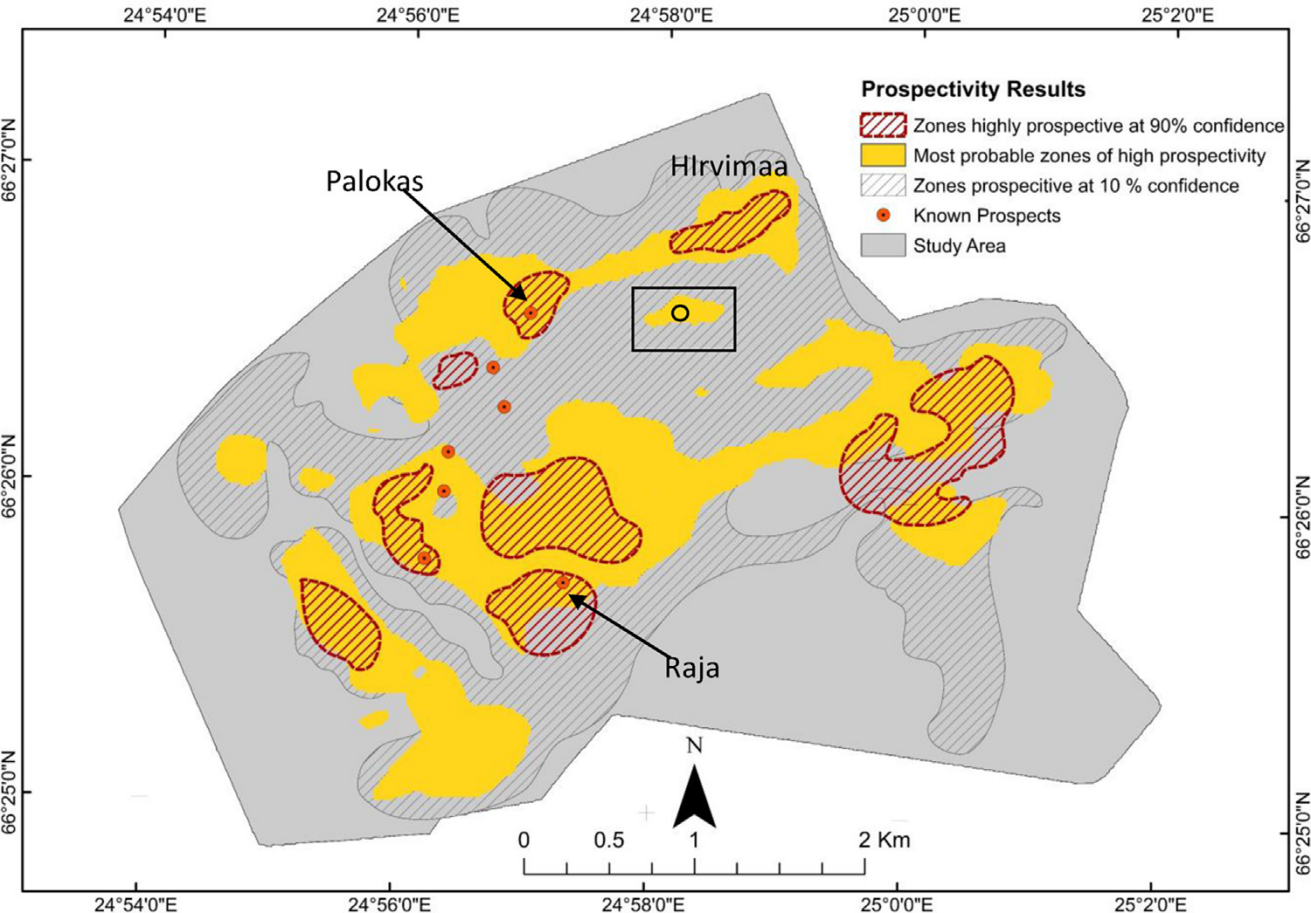
Prospectivity map derived from the FISs optimized using MCS. The search-space reduction efficiency of the FIS models is illustrated by the progressive reduction of the high prospectivity areas with increasing confidence levels.

Fig. 1 demonstrates the above-described method of parameter estimation applied to the input variable NE-SW magnetic anomalies. The input variable representing NE-SW magnetic anomalies has three linguistic values. However here we describe the parameter estimation for the membership function representing the linguistic value ‘Mineralized anomalies’. Based on the descriptive statistics in Table 1, Fig. 1a and b present the beta-PERT distribution defined for the parameters *σ, µ* of the ‘Mineralized anomalies’ membership function. From these distributions the parameters of the membership function were randomly sampled by running MCS (Fig. 1a and b, respectively). Fig. 1c shows the distribution of the output fuzzy membership values obtained from the simulated parameters. The 90% certainty ranges from the 10 percentile and 90 percentile values, indicating the possibly minimum and maximum output membership values. The parameters corresponding to the 10- and 90- percentile values were then used to define membership functions for the ‘Mineralized anomalies’ linguistic variable at 90% and 10% confidence levels, respectively (Fig. 2). This procedure was implemented for each linguistic value of each input variable. (There were a total – 17 linguistic values in 7 evidence layers – Table 2). Subsequently the parameters corresponding to the 50-, 10-, and 90- percentile output fuzzy membership values for all the variables were extracted and used to the design the expected FIS and the supplementary 90% and 10% confidence levels FISs, respectively (Fig. 3, Fig. 4). The simulated results were combined to generate the host-chemical traps and structural settings FISs at varying confidence levels (Figs. 3, 4). Finally, the fuzzy prospectivity values from each of the FISs were mapped to produce corresponding prospectivity map (Fig. 5).

**Table 2:** FIS-based modeling: Input variables, linguistic fuzzy sets, types of membership functions, membership function parameters and subjective reasoning of model parameters.

Evidence Layer (Drill core data statistics for M and NM: Q1-Median-Q3; Mean; SD)	Linguistic Fuzzy Sets	Type of Membership Function (parameters)	Parameter Values	Subjective Reasoning*
Inputs to the 'Host-Chemical Traps' FIS				
Density of magnetic tilt derivative's ‘0′ and ‘+/- ∏/4′ contours (M: 0.554–0.686–0.745; 0.654; 0.123) (NM: 0.521–0.602–0.702; 0.609; 0.136)	Low	Composite Gaussian (σ, µ) ^(i)^	σ = 0.095, µ = 0.372	Confining the 'Low' fuzzy set curve to values < Q1 values of the mineralized drill core sections.
	High	Composite Gaussian (σ, µ) ^(i)^	σ = 0.15, µ = 0.722	The 'High' fuzzy set curve covers values from the Q1 to Q3 values of the mineralized drill core sections.
AEM: In-phase to Quadrature ratio (M: 0.390–0.392–0.394; 0.391; 0.004) (NM: 0.391–0.392–0.395; 0.393; 0.005)	Low to Non-conductive units	Generalized bell function (a, b, c) ^(ii)^	*a* = 0.308, *b* = 3.411, *c* = 0.045	The degree of membership to the fuzzy set decreases gradually as the in-phase to quadrature ratio increases and reaches the Q1 values of the mineralized drill core sections.
	Conductive Mineralization	Trapezoid (a, b, c, d)^(iii)^	*a* = 0.37, *b* = 0.38, *c* = 0.393, *d* = 0.4	Confining the fuzzy set between the Q1 and Q3 values of the mineralized drill core sections, peaking around its mean-median values
	Conductive Non- Mineralized units	Composite Gaussian (σ, µ) ^(i)^	σ = 0.001, µ = 0.395	These units are mainly graphite units that show conductivity higher than the mineralized units, but do not contain mineralization (As evidenced from drill core data and discussions with field geologists).
Density of lithological contacts weighted by reactivity- OR competence- contrast (M: 0.545–0.610–0.720; 0.622; 0.137) (NM: 0.472–0.535–0.608; 0.524; 0.168)	Low	Sigmoid (a, c) ^(iv)^	*a* = −10, *c* = 0.524	Gradual decrease of the degree of membership to the fuzzy set ‘Low’ as the density value increases and consequent increase in the degree of membership to the fuzzy set ‘High’. The degree of membership to fuzzy set ‘High’ increases more steeply for values greater than the median values of the mineralized drill core sections.
	High	Composite Gaussian (σ, µ) ^(i)^	σ = 0.114, µ = 0.65	
Inputs to the 'Structural Settings' FIS				
NW-SE trending anomalies (M: 0.360–0.385–0.411; 0.386; 0.042) (NM: 0.391–0.410–0.437;0.411; 0.045)	Non-anomalous: Low	Composite Gaussian (σ, µ) ^(i)^	σ = 0.06, µ = 0.25	The degree of membership to the ‘non-anomalous-low’ fuzzy set was constrained to less than the Q1 value of mineralized drill core sections; i.e. below 0.36; the parameters of the MF were adjusted accordingly.
	Anomalous	Composite Gaussian (σ, µ) ^(i)^	σ_L_ = 0.042, µ_L_ = 0.385 σ_R_ = 0.042, µ_R_ = 0.4	The high degree of membership to the fuzzy set is centered between the median and just below Q3 of the mineralized drill core sections; the SD of the MF corresponds to that of the mineralized drill core data.
	Non-anomalous: High	Composite Gaussian (σ, µ) ^(i)^	σ = 0.01, µ = 0.676	Values close to or greater than the mean of non-mineralized drill core section have high degree of membership in this fuzzy set. (i.e. >0.40).
NE-SW trending anomalies (M: 0.186–0.211–0.240; 0.217; 0.049) (NM: 0.136–0.167–0.225; 0.191; 0.078)	Non-anomalous: Low	Composite Gaussian (σ, µ) ^(i)^	σ = 0.04, µ = 0.123	The curve is adjusted to cover values lower than median and grading to the mean of non-mineralized drill core sections.
	Anomalous	Gaussian (σ, µ) ^(i)^	σ = 0.04, µ = 0.215	The curve is adjusted to cover values between the mean and median of the mineralized drill core sections.
	Non-anomalous: High	Sigmoid (a, c) ^(iv)^	*a* = 60, *c* = 0.281	The MF assigns high degree of membership to values > Q3 value of the mineralized drill core sections.
Density of gravity worms (M: 0.586–0.7–0.811; 0.682; 0.136) (NM: 0.250–0.444–0.606; 0.441; 0.220)	Low	Sigmoid (a, c) ^(iv)^	*a* = −7.605, *c* = 0.432	A gently sloping MF extending till the Q3 value of non-mineralized drill core sections.
	High	Composite Gaussian (σ, µ) ^(i)^	σ = 0.149, µ = 0.75	For mineralized drill core sections, the mean 〈 median; this curve is fitted such as to include values 〉 mean in the fuzzy set.
Density of faults weighted by their sinuosity or structural intersection zones. (M: 0.281–0.375–0.482;0.410;0.180) (NM: 0.212–0.287–0.418; 0.313; 0.152)	Low	Composite Gaussian (σ, µ) ^(i)^	σ = 0.12, µ = 0.2	The curve assigns high degree of membership for values up to the median of non-mineralized drill core sections, after that the degree of membership decreases gradually till Q3.
	High	Composite Gaussian (σ, µ) ^(i)^	σ = 0.168, µ = 0.54	The curve assigns high degree of membership to values greater than the median values of the mineralized drill core sections.

Abbreviations: AEM = Airborne Electromagnetics; MF = Membership Function; M = Mineralized drill core sections; NM = Non mineralized drill core sections; Q1= 1st Quartile; Q2 = 2nd Quartile; Q3 = 3rd Quartile; Q4 = 4th Quartile; SD = Standard deviation.

* Subjective reasoning formed the basis for generating the initial FIS which was then optimised using parameters obtained from MCS. Such reasoning varies from person to person and therefore lacks precision and accuracy. Nevertheless, it is important to document and make these available for the information of end-users and relevant stakeholders.

^(i)^Gaussian MF: It is defined by the parameters center (µ) and spread (σ) as follows:.

$f\left(x;\;\sigma ,\;\mu \right)=e^{\left\{-\frac{{\left(x-\mu \right)}^{2}}{2{\left(\sigma \right)}^{2}}\right\}}.$

Composite Gaussian MF: It comprises two Gaussian functions, i.e. the left and the right curves. The composite gaussian function is defined by the center (µ) and spread (σ) parameters of the left and right curves as follows:.

$f_{cg}\left(x;\;\sigma_{L},\;\mu_{L},\;\;\sigma_{R},\;\mu_{R}\right)=\;f_{L}\left(x;\;\sigma_{L},\;\mu_{L}\right)\;X\;f_{R}\left(x;\;\sigma_{R},\;\mu_{R}\right).$

where, $f_{L}\left(x;\;\sigma_{L},\;\mu_{L}\right)=\;e^{\left\{-\frac{{\left(x-\mu_{L}\right)}^{2}}{2{\left(\sigma_{L}\right)}^{2}}\right\}},\;for\;x\leq \;\mu_{L},\;else\;f_{L}\left(x;\;\sigma_{L},\;\mu_{L}\right)=1.$

$f_{R}\left(x;\;\sigma_{R},\;\mu_{R}\right)=\;e^{\left\{-\frac{{\left(x-\mu_{R}\right)}^{2}}{2{\left(\sigma_{R}\right)}^{2}}\right\}},\;for\;x\geq \;\mu_{R},\;else\;f_{R}\left(x;\;\sigma_{R},\;\mu_{R}\right)=1.$

In the composite function for µ_L_ ≤ µ_R_ the membership values reach maximum of 1 over the range [µ_L_, µ_R_]. For µ_L_ > µ_R_, the maximum value is less than 1. The composite gaussian function applied to fuzzy sets such as ‘Low’ or ‘Non-anomalous’ linguistic labels is comprised of the left curve only and when applied to fuzzy sets such as ‘High’ or ‘Anomalous’ linguistic labels it is comprised of the right curve only. The main utility of composite gaussian function is that the left and right need not be symmetric curves. This facilitates shaping the function with different slopes on either side.

^(ii)^Generalized bell function: This function is defined by parameters *a, b,* and *c* as follows:.

$f\left(x;\;a,\;b,\;c\right)=\frac{1}{1+\;{\left|\frac{x-c}{a}\right|}^{2b}}.$

where, a, b and c define the width, shape, and center of the function, respectively. At *x = (c – a)* and *x = (c + a)* the degree of membership, i.e. *f(x) =* 0.5. The width of the curve at *f(x) =* 0.5 is twice the absolute value of parameter *a*. The parameter *b* controls the slope of the transition of the membership values. Larger values of *b* cause steeper transitions. The generalized bell-shaped function is symmetric about the line *x* *=* *c.*

^(iii)^Trapezoid MF: This function is defined by the parameters *a, b, c,* and *d*, that correspond to the *x* values of the corners of the trapezoid.

$f\left(x;a,\;b,c,\;d\right)=\max\left(min\left(\frac{x-a}{b-a},\;1,\frac{d-x}{d-c}\right),\;0\right).$

*b* and *c* define the shoulder values for the central plateau region of the trapezoid, while *a* and *d* define the base of the trapezoid, thereby controlling the slope of the transition.

^(iv)^Sigmoid MF: This function can be represented as:

$f\left(x;a,\;c\right)=\frac{1}{1+\;e^{-a\left(x-c\right)}}.$

The parameters *a* and *c* control the width and center of the transition area, respectively. *c* is the inflection point where *f(x) =* 0.5 and *a* is the slope at *c*.

## Results and method validation

The FISs at varying confidence levels were derived from the 90-, 50-, and 10- percentile values of the parameters extracted from the 90% certainty envelope of probability distribution of the simulations. The consequent changes in the fuzzy membership values can be visualized for instance in Fig. 2 that shows the fuzzy membership function expected from data statistics and the supplementary membership functions at 10 and 90% confidence levels for the fuzzy set representing ‘mineralized anomalies’ in the evidence layer of NE-SW trending magnetic response. An input value attains three fuzzy membership values, one from each of the confidence levels functions. For instance, an input value 0.218 (around the peak of all the three membership functions) is mapped to nearly maximum membership value (i.e., ∼1) at 10%-, expected, and 90%- confidence levels. However, the differences in the membership values increase towards the tail of the functions, thus indicating progressive increase of the uncertainties. Additionally, for the given example, these differences are not symmetric along both the sides of the curves. For an input value of 0.25, that plots on the right-side curves, the membership values at 10%-, expected, and 90%- confidence levels are 0.825, 0.658, and 0.525, respectively. The right side of the curve shows less difference between the expected and 90%- confidence level membership values. Similarly, for an input value of 0.18, that plots on the left-side curves, the membership values at 10%-, expected and 90%- confidence levels are 0.725, 0.658, and 0.458, respectively. The left-side of the curves show relatively less differences between the expected and 10% confidence level membership values, particularly for values above the inflection points. The same input value, therefore, gets mapped to different output space at varying confidence levels.

In Figs. 3 and 4, an input feature vector with values 0.5 in all input variables has the host potential as 0.1, 0.07 and 0.04 and the favorable structural settings potential as 0.163, 0.153 and 0.122 in 10%-, expected and 90% -confidence level FISs, respectively. Hence the final prospectivity values attained by such an input feature vector ranges from 0.016 - 0.005 at 90% certainty, while the most probable prospectivity value is 0.010. The overall standard deviation of the prospectivity values for this input feature vector is 0.0055. The consequent changes in the final prospectivity values when all the variables are integrated in the 10%-, expected and 90%- confidence level FISs are evident in the prospectivity results also (Fig. 5). As the confidence level of FIS results increases from 10% to 90% the area mapped as highly prospective reduces spatially and the high prospectivity zones become well-defined and localized (Fig. 5). For instance, a random location marked by ‘o’ in the black box in Fig. 5 has prospectivity values ranging from 0.806 to 0.167 at 10–90%- confidence levels, respectively (standard deviation of 0.26, implying high uncertainty). In the Hirvimaa area, or the well-endowed Palokas and Raja prospects the prospectivity is enhanced and the spatial extent of prospective zones is reduced as the confidence level increases from 10 to 90% confidence. Such variations in the prospectivity values therefore facilitate identification of exploration targets with a confidence factor attached to the results. Hence, we were able to quantify the model uncertainties by assigning confidence level to the reported results.

**Fig. 6:**
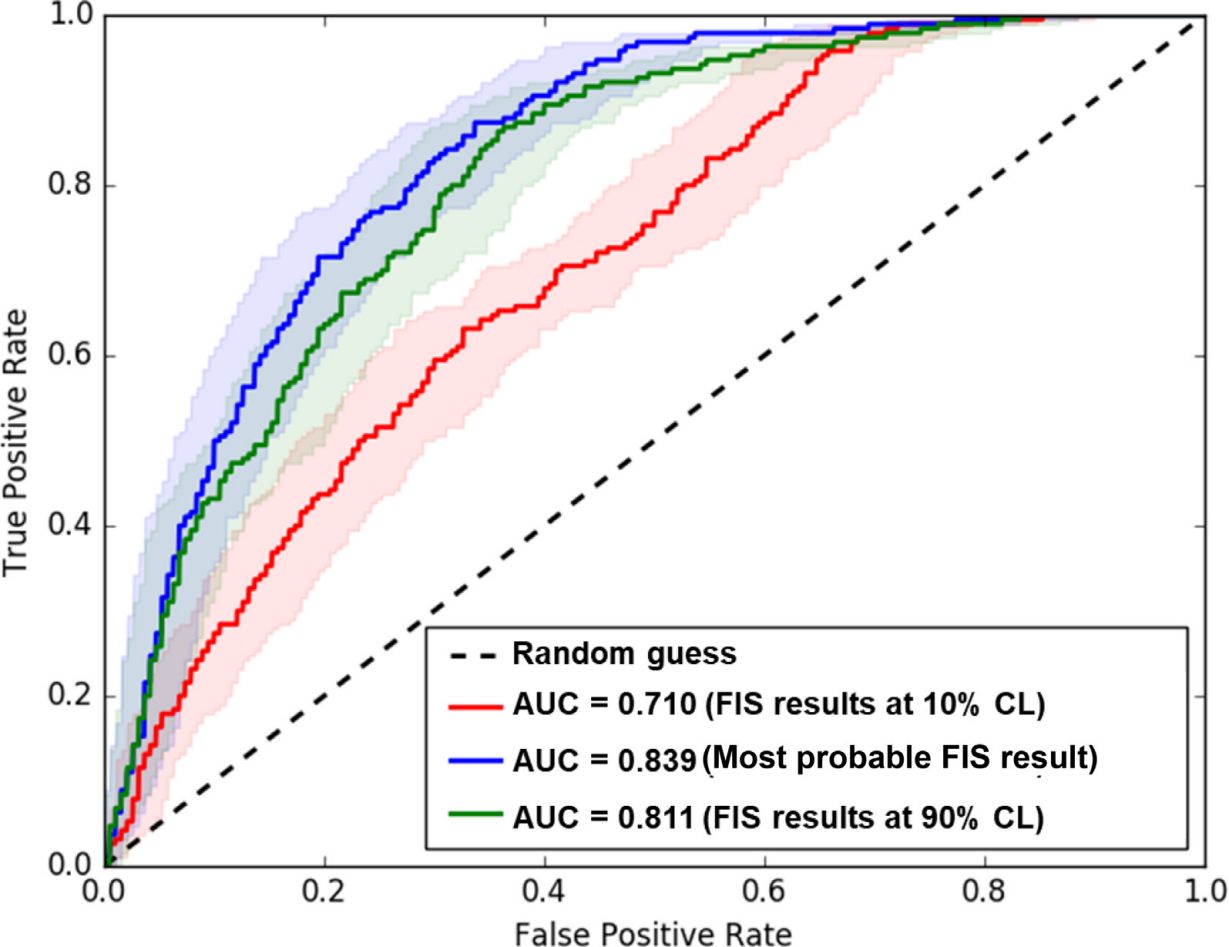
Validation of FIS classification results using ROC plots. All three confidence level FIS results show an overall high AUC value. The shaded area shows the 95% confidence level region for the AUC values of the respective curve. (Reproduced from [2]).

Additionally, all the results are validated using the receiver operating characteristics (ROC) curves and the corresponding area under curve (AUC) values (Fig. 6). The prospectivity map from the optimized FIS shows AUC value of 0.839 and the supplementary results also have high AUC values of 0.710, and 0.811 for the 10%-, and 90%- confidence level results, respectively. The capture efficiency, tabulated in Table 3, further validate the efficiency of the method proposed in this study. The FIS model captures 80% of the mineralized drill core sections in the highly prospective tracts that occupy 24% of the study area. The ground exploration targets identified by the prospectivity maps together comprise about 1.5 km^2^ of the ∼20 km^2^ study area (i.e., 7.5% of the study area) [2]. This indicates considerable search-space reduction by the modeling results for follow-up ground exploration with high confidence.

**Table 3:** FIS model results, statistics, and capture efficiency. The FIS model captures 80% of the mineralized drill core sections in the highly prospective tracts that occupy 24% of the study area. (reproduced from [2]).

Prospectivity Class	10% Confidence Level Results			50% Confidence Level Results			90% Confidence Level Results		
	Prospectivity value range	% mineralized locations	% Study Area	Prospectivity value range	% mineralized locations	% Study Area	Prospectivity value range	% mineralized locations	% Study Area
Very High	0.80–1.00	3	1.5	0.82–1.00	38	4	0.81–1.00	12	2
High	0.60–0.80	22	8.5	0.53–0.82	42	20	0.58–0.81	36	7
Average	0.20–0.60	68	45	0.30–0.53	18	24	0.19–0.58	42	29
Low	0.00–0.20	7	45	0.13–0.30	2	20	0.01–0.18	10	46
Very Low	–	–	–	0.00–0.15	0	32	0.00–0.01	0	14

## Declaration of Competing Interest

No competing interests to declare.
